# Is There an Association Between Living in a Rural Area and the Incidence of Postoperative Complications or Hospital Readmissions Following Left Ventricular Assist Device (LVAD) Implantation, Compared to Urban Lvad Recipients? A Systematic Review

**DOI:** 10.1002/clc.70068

**Published:** 2025-01-01

**Authors:** Samrat Gollapudi, Abhiram Gollapudi, Sri Banala, Sheraj Singh, Kiran Tadi

**Affiliations:** ^1^ Department of Medicine Rowan School of Osteopathic Medicine Stratford New Jersey USA; ^2^ Department of Research Future Forwards Research Institute Piscataway New Jersey USA

**Keywords:** adverse event, left ventricular‐assist device, LVAD, mechanical circulatory support, morbidity, mortality, rehospitalization, rural versus urban, VAD, ventricular assist device

## Abstract

**Background:**

Left ventricular assist devices (LVADs) are utilized as a therapeutic option for patients with end‐stage heart failure. While LVAD implantation can enhance survival rates and quality of life, the procedure has its risks, and postoperative complications are common. This review aims to investigate whether there is an association between living in a rural area and the incidence of postoperative complications or hospital readmissions following LVAD implantation, compared to urban LVAD recipients.

**Methods:**

A comprehensive literature review examined studies that compared postoperative outcomes between rural and urban LVAD recipients. Data on adverse events, hospitalizations, and mortality rates were extracted, focusing on the impact of geographic location on these outcomes.

**Results:**

The review found that rural LVAD recipients may be at a higher risk for certain complications, including gastrointestinal bleeding, ventricular arrhythmias, LVAD complications, and stroke. Rural patients also exhibited higher instances of emergency department visits and hospital readmissions. Despite these challenges, survival rates and heart transplantation outcomes at 1 year were similar between rural and urban recipients. However, rural patients exhibited a higher driveline infection rate at 1 year.

**Conclusion:**

The findings of this review suggest that rural residency may be associated with an increased risk of certain postoperative complications and hospital readmissions following LVAD implantation. These results highlight the need for healthcare strategies to address the challenges faced by rural LVAD recipients. Further research is necessary to understand the relationship between geographic location and LVAD outcomes and to develop interventions that can improve postoperative care for this vulnerable population.

## Graphical abstract

Left ventricular assist device (LVAD) recipients show distinct outcome patterns based on geographic location. Rural patients face unique challenges including longer distances to treatment centers and higher emergency department utilization. While both populations maintain similar 1‐year survival rates (82%), rural recipients demonstrate higher rates of specific complications, including gastrointestinal bleeding, ventricular arrhythmias and stroke. These findings highlight the need for targeted interventions to address healthcare disparities while maintaining the overall effectiveness of LVAD therapy across geographic locations.

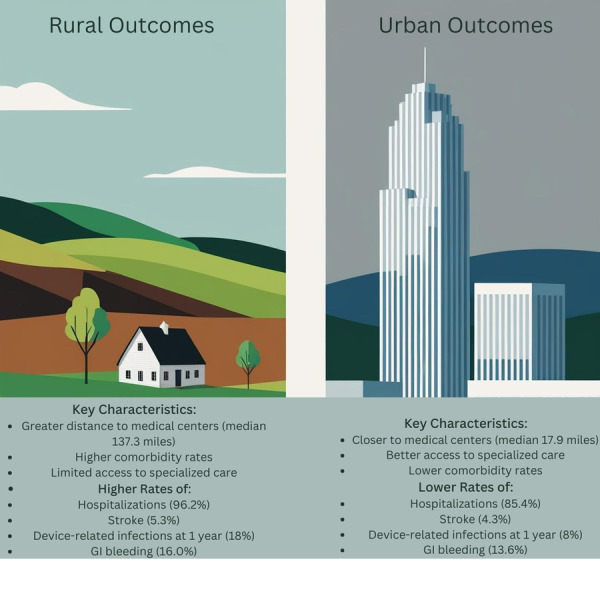

## 1 Introduction

A left ventricular assist device (LVAD), commonly called LVAD, is a battery‐powered device that aids in pumping blood out of the lower left ventricle to the rest of the body. Although initially used as a temporary treatment for acute heart failure in patients waiting for transplants, LVADs are now commonly used as treatment options for heart failure, especially in patients with comorbidities preventing transplantation [1]. One of the goals of LVAD treatment is termed “reverse remodeling,” or improved lower chamber volumes, heart rate responsiveness, and β‐adrenergic responsiveness [2, 3]. The mechanics of LVADs continuously evolve to improve patient outcomes; however, the core function remains the same for both continuous and pulsatile flow devices [4]. The inflow cannula portion of the LVAD is attached to the left ventricular apex. Blood from the lungs enters the left side of the heart, where the LVAD device pumps the blood through the outflow canal into the ascending aorta, which is then distributed to the rest of the body. The pump and battery are controlled outside the body, and the driveline portion enters the skin to control the device [5, 6]. LVADs are particularly helpful in patients with end‐stage heart failure. In the United States, over 6.5 million people have heart failure, and this number continues to grow [7]. Due to lengthy transplant lists and organ availability, LVADs have become a mainstay for maintaining heart function in patients waiting for transplants or patients who have tried multiple rounds of medical therapy. Patients who have received a CF‐LVAD are shown to have a 1‐year survival of 84%. This is a substantial increase in comparison to the 53% 2‐year survival rate in heart failure patients on medical therapy [8]. Despite increased patient and physician education and advancements in the actual LVAD mechanics, up to 60% of patients will experience an LVAD‐related complication by 6 months post‐surgery [9]. Complications of LVAD implantation are well documented and studied. Approximately 50‐85% of patients experience bleeding requiring blood transfusion, 30% of patients experience bleeding requiring reoperation, and 50% of patients will contract infections [10]. Additionally, there is a 2%–9% rate of pump thrombosis that may lead to cardiogenic shock, a 15%–25% rate of right heart failure, and a 10%–15% rate of stroke [11, 12, 13]. Ventricular arrhythmias within the 30‐day postoperative window are also common, with an incidence rate between 22% and 59% [14, 15]. As LVAD implantation rates continue to grow, it is increasingly important that complications are monitored in all capacities. To date, few studies have examined the epidemiological factors affecting complication rates due to LVAD transplantation. Urban populations, areas containing 50 000 or more people or clusters of at least 2500 people, have greater access to medical services and physicians [16, 17]. In contrast, rural populations, areas that do not meet urban criteria, are more likely to experience health problems and are less likely to have medical services available to them [17]. This review compares rates of adverse effects due to LVAD implantation in rural hospitals versus urban hospitals.

## 2 Search Terms

Key terms used to identify studies pertinent to the above research question include: (“left ventricular assist device” OR “left ventricular‐assist device” OR “LVAD” OR “ventricular assist device” OR “ventricular‐assist device” OR “VAD” OR “mechanical circulatory support” OR “MCS”) AND (“morbidity” OR “mortality” OR “rehospitalization” OR “quality of life” OR “significant adverse event” OR “adverse event” OR “prevalence” OR “incidence”) AND (“rural” OR “urban”). An initial article search was conducted on 10‐21‐2023. Duplicate studies were removed using Rayyan.ai software, followed by a manual review to remove any additional copies. The following databases were used for our search: PubMed, Embase, Scopus, Web of Science, and Cochrane Library.

## 3 Inclusion and Exclusion Criteria

Articles published within the last 10 years (from 2014 to 2014) involve LVAD (Left Ventricular Assist Device) patients, studies that compare outcomes (e.g., adverse events, mortality) between rural and urban populations, and studies that report specific outcomes of interest (e.g., adverse events, mortality) in LVAD patients were included. Articles were excluded if they did not include rural and urban populations, if they did not report specific outcomes of interest (e.g., adverse events, mortality) in LVAD patients, or if said studies were published more than 10 years ago.

## 4 Data Collection and Selection Process

**Figure 1:**
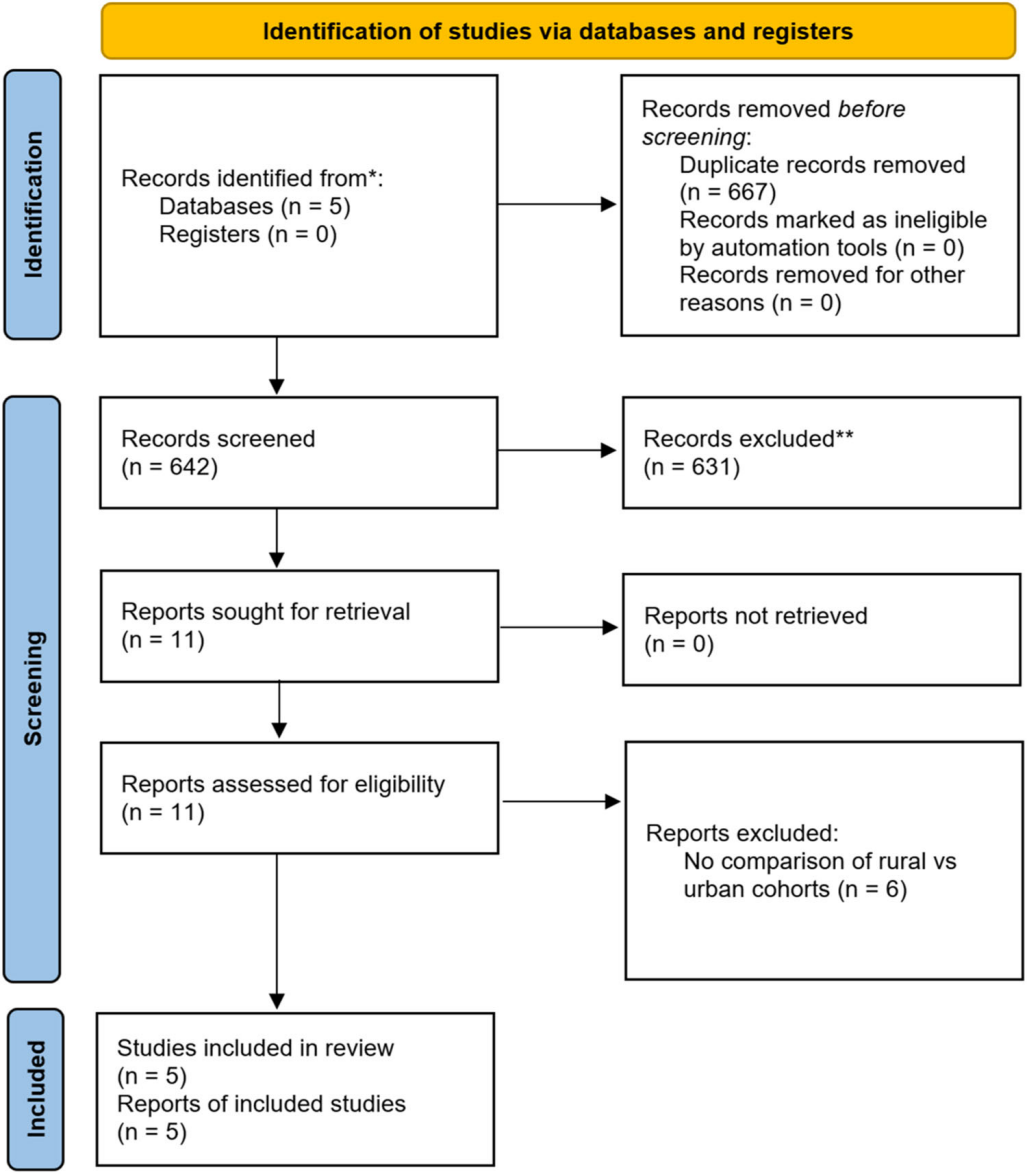
PRISMA 2020 flow diagram for study selection and data collection.

This systematic review was conducted using Preferred Reporting Items for Systematic Reviews and Meta‐Analyses (PRISMA 2020) guidelines. Duplicates were removed. Two reviewers completed the title and abstract review followed by a full‐text appraisal. If no conclusion was reached, a third reviewer was involved to break the tie. Figure 1 shows the PRISMA flow diagram detailing our study selection process.

## 5 Certainty of Evidence and Risk of Bias Assessment

The quality of evidence for each outcome was assessed using the Grading of Recommendations Assessment, Development, and Evaluation (GRADE) criteria. For this systematic review, two reviewers applied the GRADE criteria independently to assess the quality of evidence for each outcome. Any discrepancies were resolved through discussion and consensus. The risk of bias in the included studies was assessed using the Cochrane Risk of Bias tool for randomized controlled trials (RCTs) and the Newcastle‐Ottawa Scale (NOS) for non‐randomized studies. Two reviewers independently assessed the risk of bias in each included study. Any disagreements were resolved through discussion and consultation with a third reviewer if necessary. The risk of bias assessment results were used to inform the overall quality of evidence for each outcome according to the GRADE criteria.

## 6 Results

**Figure 2:**
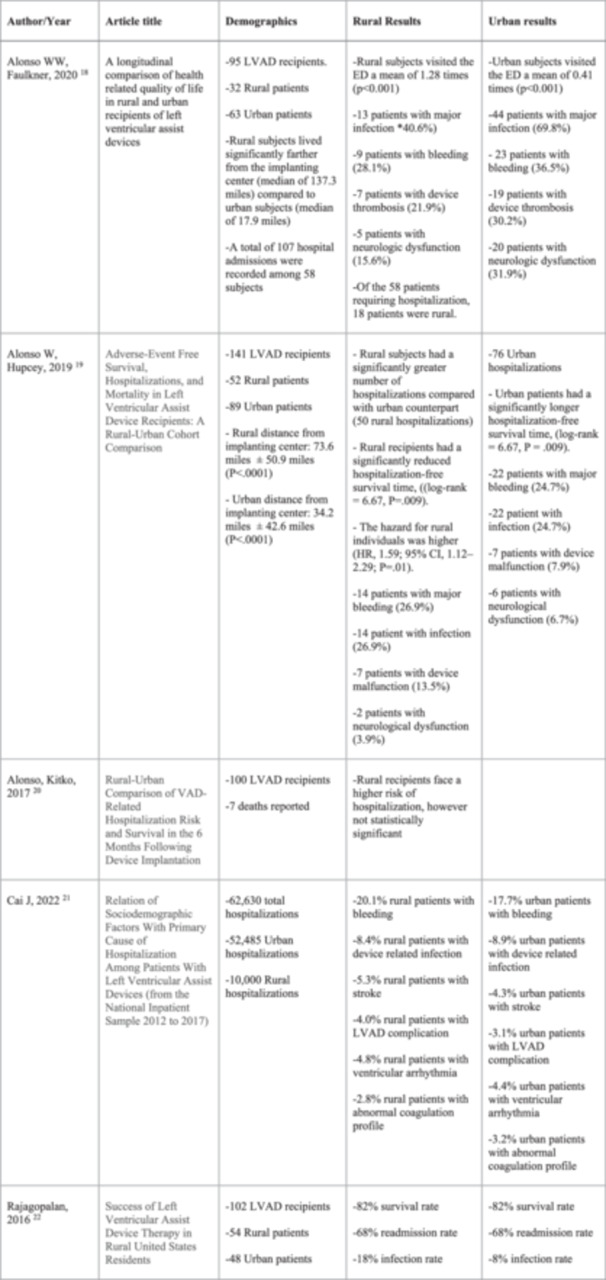
Comparing adverse events and hospitalizations between Rural and Urban LVAD recipients.

Several studies have been conducted evaluating the relationship between rural and urban LVAD recipients and their association with adverse events/hospitalizations. Data from a retrospective cohort study involving 141 patients was analyzed, with 52 classified as rural and 89 as urban based on their county of residence. This study revealed that two‐thirds of LVAD recipients (94/141, 68.1%) experienced at least one adverse event with bleeding being the most common event (26.9% rural vs 24.7% urban, *p* = 0.37). This was followed by infection (26.9% rural vs. 24.7% urban, *p* = 0.37), device malfunction (13.5% rural vs 7.9% urban, *p* = 0.83), and neurological dysfunction (3.9% vs 6.7%, *p* = 0.74). Figure 2 provides a comparative summary of adverse events and hospitalization rates between rural and urban LVAD recipients, highlighting witnessed differences. Interestingly, these events did not significantly differ between rural and urban recipients (*p* = 0.28). However, when evaluating hospitalizations, rates were significantly higher among rural LVAD recipients, with 96.2% of rural participants experiencing at least one hospitalization compared to 85.4% of urban participants (*p* = 0.04). This study used a Cox proportional hazard model to evaluate mortality between the rural and urban groups, this model showed no significant difference in all‐cause mortality between rural and urban groups (hazard ratio [HR], 0.45; 95% confidence interval [CI], 0.17–1.23; *p* = 0.12). However, rural recipients had a shorter hospitalization‐free survival period compared to their urban counterparts [18].

In a different study by Kitko, 100 VAD recipients discharged from the hospital following implantation were followed during the 6‐month study period. Over 60% of recipients experienced at least one hospitalization, with seven recipients' deaths. When analyzing the data, Kaplan–Meier curves comparing hospitalizations suggested that rural VAD recipients may be at a higher hospitalization risk than urban recipients. Furthermore, Cox proportional hazard models were used to compare the hazard ratios for mortality and hospitalization between rural and urban VAD recipients. The models did not find statistically significant differences in mortality or hospitalization risk between the two groups [19].

Faulker's analysis of data taken from the “Profiling Biobehavioral Responses to Mechanical Circulatory Support in Advanced Heart Failure” (PREMISE) study showed 97% of the involved patients experienced at least one adverse event within the 6‐month study period, with no significant differences between rural and urban recipients. The most common adverse events were major infection (57 events, with 23% occurring in rural subjects), bleeding (32 events, with 28% in rural subjects), device thrombosis (26 events, with 27% in rural subjects), and neurologic dysfunction (25 events with 20% occurring in rural subjects). Notably, out of the 95 included subjects, 32 (33.7%) were living in rural areas, and 63 (66.3%) were in urban areas. Moreover, rural subjects had significantly higher rates of comorbidities such as myocardial infarction, hypertension, and higher mean body mass index (BMI) compared to urban subjects. The median distance from the implanting center was also significantly greater for rural subjects at 137.3 miles compared to 17.9 miles for urban subjects. No statistically significant differences in hospitalizations were seen despite the greater distance between the rural and urban subjects. Of 58 subjects requiring hospitalizations, 18 patients were from rural areas [20].

Cai's (2022) comprehensive data analysis on hospitalizations from 2012 to 2017 showed several critical findings on the incidence of postoperative complications and readmissions among patients residing in rural versus urban areas. Regarding cardiovascular complications, stroke was notably more common among patients from rural areas compared to their urban counterparts (5.3% vs 4.3%). Likewise, ventricular arrhythmias (4.8% rural vs 4.4% urban) and LVAD complications (4.0% rural vs 3.1% urban) showed a similar difference between the two groups. Regarding non‐cardiovascular complications, gastrointestinal bleeding was a prevalent issue, occurring more frequently in rural patients than urban patients (16.0% vs 13.6% respectively). Incidence of device‐related infections or sepsis also showed a trend towards higher rates in urban settings (8.9% urban vs 8.4% rural). rates of hospital readmissions within 30 days post‐LVAD implantation. The results indicated a higher likelihood of readmission for patients residing in rural areas [21].

Rajagopalan's study evaluated 102 patients who were separated into two groups: rural (*n* = 54, 53%) and urban (*n* = 48, 47%). Between both cohorts, the infection rate at 1 year was higher in the rural population (18% rural vs 8% urban). Survival in 1 year was the same, at 82% across both groups [22].

**Figure 3:**
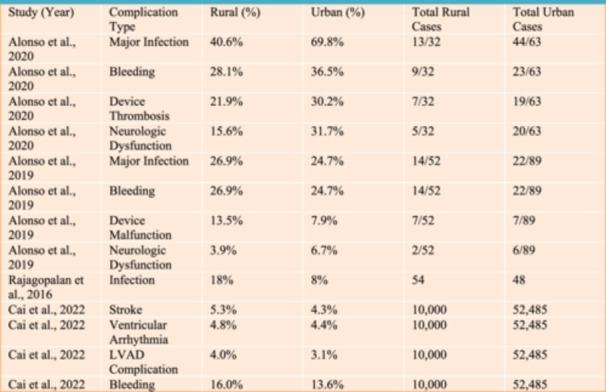
Complication Rates Across LVAD Studies: Rural versus Urban.

Complication rates, categorized across the included studies, are detailed in Figure 3, showcasing variability in outcomes between the two populations.

## 7 Discussion

Our systematic review explores the association between complications following the insertion of LVAD devices and postoperative complications in rural versus urban areas. Through the review of numerous peer‐reviewed research articles, we have compiled evidence that points to a significant difference.

While certain complications such as infection, bleeding, device malfunction, and neurological dysfunction did not significantly differ between rural and urban LVAD recipients, the rate of hospitalization was significantly higher in rural patients. Elevated rates of stroke and gastrointestinal bleeding were also observed. It is important to note the definitive discrepancies in access to healthcare between rural and urban populations. Larger travel distances to medical facilities often result in delayed treatment, while elevated healthcare costs can make crucial follow‐up appointments prohibitively expensive. These communities also struggle with a persistent shortage of healthcare providers, with general practitioners often being their primary, and sometimes only, source of medical care. The situation is further compounded by a lack of cardiac specialists, despite the need for specialized management in these areas. Additionally, rural hospitals typically operate with limited services and capabilities, further compromising the comprehensive care these patients need [23, 24]. All of these challenges are particularly concerning as rural populations not only present with higher rates of pre‐implantation comorbidities but also face increased risks from conditions like vascular disease, obesity, diabetes, and hypertension. These health disparities, alongside an aging rural population, illustrate the existing obstacles in providing adequate cardiac care to these communities [23, 25]. Tailored treatment and prevention plans are neccessary post‐LVAD placement according to the patient demographic.

Our review is grounded in exploring various methodologies and research styles, yet we are cognizant of the limitations that observational studies may include, such as the reliance on retrospective studies and associated biases. We also acknowledge the heterogeneity of LVAD populations (ie HMII vs. HVAD vs. HMIII), and the significant differentials in survival and adverse events between these pumps. Future research endeavors should dive deeper into the underlying factors contributing to disparities in postoperative outcomes between rural and urban LVAD recipients. Importantly, over the last 10 years, LVAD technology has evolved with generally fewer adverse LVAD events—therefore, for future studies, it may be advantageous to consider subtype of LVAD or report incidences of adverse LVAD outcomes based on era of LVAD implant (ie 2010–2015, 2015–2020, post 2020 etc). This may necessitate a comprehensive exploration of healthcare infrastructure, access to specialized care, socioeconomic determinants, and patient demographics. Finally, interventions, such as telemedicine initiatives, outreach programs, and healthcare reform policies, should be advocated for and implemented. This can ensure ameliorated outcomes and equal access for all LVAD recipients, irrespective of geographic location.

## 8 Conclusion

This systematic review highlights the need for investigating socioeconomic and geographic disparities in healthcare access, specifically in the case of LVAD recipients in rural and urban settings. We recognize the need for targeted interventions and strive to implement such interventions head‐on to make equal yet personalized care a standard in all settings.

## Ethics Statememt

This study is a systematic review and did not involve direct research with human participants or animals. Therefore, Institutional Review Board (IRB) approval was not required. The included studies in the review adhered to ethical standards, as stated in their original publications.

## Conflicts of Interest

The authors declare no conflicts of interest.

## Data Availability

Data sharing not applicable to this article as no datasets were generated or analyzed during the current study. The datasets analyzed during the current study are derived from publicly accessible databases, including PubMed, Embase, Scopus, Web of Science, and the Cochrane Library. Specific details regarding the search terms, inclusion and exclusion criteria, and the systematic review methodology are provided in the manuscript. Further information or clarifications about the data used in the study can be requested from the corresponding author.

## References

[1] E. E. Adams and M. L. Wrightson , “Quality of Life With an LVAD: A Misunderstood Concept,” Heart & Lung 47, no. 3 (2018): 177–183, 10.1016/j.hrtlng.2018.02.003. 29551363

[2] N. Koitabashi and D. A. Kass , “Reverse Remodeling in Heart Failure‐‐Mechanisms and Therapeutic Opportunities,” Nature Reviews Cardiology 9, no. 3 (2011): 147–157, 10.1038/nrcardio.2011.172. 22143079

[3] J. Wohlschlaeger , N. Kawaguchi , B. Levkau , C. Schmid , H. H. Scheld , and H. A. Baba , “[Left Ventricular Assist Devices (LVAD): Optional Treatment Alternative to Cardiac Transplantation?],” Verhandlungen der Deutschen Gesellschaft fur Pathologie 88 (2004): 113–121. 16892541

[4] S. Kadakia , R. Moore , V. Ambur , and Y. Toyoda , “Current Status of the Implantable LVAD,” General thoracic and cardiovascular surgery 64, no. 9 (2016): 501–508, 10.1007/s11748-016-0671-y. 27270581

[5] S. P. Chaudhry , A. D. DeVore , H. Vidula , et al., “Left Ventricular Assist Devices: A Primer For the General Cardiologist,” Journal of the American Heart Association 11, no. 24 (2022): e027251, 10.1161/JAHA.122.027251. 36515226 PMC9798797

[6] Y. Vaidya , S. Riaz , and A. S. Dhamoon , “Left Ventricular Assist Devices. [Updated August 8, 2023].” StatPearls [Internet] (Treasure Island (FL): StatPearls Publishing, 2024 Jan). 29763016

[7] C. Chen‐Scarabelli , L. Saravolatz , B. Hirsh , P. Agrawal , and T. M. Scarabelli , “Dilemmas in End‐Stage Heart Failure,” Journal of Geriatric Cardiology: JGC 12, no. 1 (2015): 57–65, 10.11909/j.issn.1671-5411.2015.01.007. 25678905 PMC4308459

[8] O. Wever‐Pinzon , S. G. Drakos , A. G. Kfoury , et al., “Morbidity and Mortality in Heart Transplant Candidates Supported With Mechanical Circulatory Support: Is Reappraisal of the Current United Network for Organ Sharing Thoracic Organ Allocation Policy Justified?,” Circulation 127, no. 4 (2013): 452–462, 10.1161/CIRCULATIONAHA.112.100123. 23271796 PMC3752367

[9] A. V. Ambardekar , M. M. Kittleson , M. Palardy , et al., “Outcomes With Ambulatory Advanced Heart Failure From the Medical Arm of Mechanically Assisted Circulatory Support (Medamacs) Registry,” Journal of Heart and Lung Transplantation 38 (2019): 408–417, 10.1016/j.healun.2018.09.021. PMC6452871 30948210

[10] P. M. McCarthy , S. K. Schmitt , R. L. Vargo , S. Gordon , T. F. Keys , and R. E. Hobbs , “Implantable LVAD Infections: Implications for Permanent Use of the Device,” Annals of Thoracic Surgery 61, no. 1 (1996): 359–365, 10.1016/0003-4975(95)00990-6. 8561605

[11] A. Kilic , M. A. Acker , and P. Atluri , “Dealing With Surgical Left Ventricular Assist Device Complications,” Journal of Thoracic Disease 7, no. 12 (2015): 2158–2164, 10.3978/j.issn.2072-1439.2015.10.64. 26793336 PMC4703654

[12] J. Goodman and S. Lerakis , “The Aortic Valve: The Gatekeeper of the LVAD,” CASE 4, no. 5 (2020): 341–342, 10.1016/j.case.2020.05.015. 33117924 PMC7581613

[13] D. Acharya , T. Kazui , D. Al Rameni , et al., “Aortic Valve Disorders and Left Ventricular Assist Devices,” Frontiers in Cardiovascular Medicine 10 (2023): 1098348, 10.3389/fcvm.2023.1098348. 36910539 PMC9996073

[14] A. Ahmed , M. Amin , B. A. Boilson , A. M. Killu , and M. Madhavan , “Ventricular Arrhythmias in Patients With Left Ventricular Assist Device (Lvad),” Current Treatment Options in Cardiovascular Medicine 21, no. 11 (2019): 75, 10.1007/s11936-019-0783-7. 31773322

[15] Z. Jedeon , R. Cogswell , J. Schultz , L. Von Wald , R. John , and H. Roukoz , “Association Between Early Ventricular Arrhythmias and Mortality in Destination Vs. Bridge Patients on Continuous Flow Lvad Support,” Scientific Reports 11, no. 1 (2021): 19196, 10.1038/s41598-021-98109-2. 34584108 PMC8479086

[16] *Defining Rural at the U.S. Census Bureau*, www2.census.gov/geo/pdfs/reference/ua/Defining_Rural.pdf.

[17] X. Chen , H. Orom , J. L. Hay , et al., “Differences in Rural and Urban Health Information Access and Use,” Journal of Rural Health 35, no. 3 (2019): 405–417, 10.1111/jrh.12335. PMC6522336 30444935

[18] W. W. Alonso , K. M. Faulkner , B. J. Pozehl , J. E. Hupcey , L. A. Kitko , and C. S. Lee , “A Longitudinal Comparison of Health‐Related Quality of Life in Rural and Urban Recipients of Left Ventricular Assist Devices,” Research in Nursing & Health 43, no. 4 (2020): 396–406, 10.1002/nur.22052. 32627852 PMC7500868

[19] W. Alonso , J. E. Hupcey , L. Kitko , B. Pozehl , and K. Kupzyk , “Adverse‐Event Free Survival, Hospitalizations, and Mortality in Left Ventricular Assist Device Recipients: A Rural‐Urban Cohort Comparison,” Journal of Cardiovascular Nursing 34, no. 6 (2019): 454–464, 10.1097/JCN.0000000000000597. 31365445 PMC8381739

[20] W. Alonso , “Abstract 20156: Rural‐Urban Comparison of VAD‐Related Hospitalization Risk and Survival in the 6 Months Following Device Implantation,” Circulation 136 (2017): 44, https://www.ahajournals.org/doi/10.1161/circ.136.suppl_1.20156.

[21] J. Cai , W. Xia , P. Greenberg , I. Okwuosa , S. Setoguchi , and E. Akhabue , “Relation of Sociodemographic Factors With Primary Cause of Hospitalization Among Patients With Left Ventricular Assist Devices (From The National Inpatient Sample 2012 to 2017),” American Journal of Cardiology 180 (2022): 81–90, 10.1016/j.amjcard.2022.06.047. 35945042

[22] N. Rajagopalan , A. L. Hart , and S. M. Branam , Success of Left Ventricular Assist Device Therapy in Rural United States Residents, https://www.embase.com/records?subaction=viewrecord&id=L72253847.

[23] L. G. Hart , E. H. Larson , and D. M. Lishner , “Rural Definitions for Health Policy and Research,” American Journal of Public Health 95, no. 7 (2005): 1149–1155, 10.2105/ajph.2004.042432. 15983270 PMC1449333

[24] T. S. Gruca , T. H. Pyo , and G. C. Nelson , “Providing Cardiology Care in Rural Areas Through Visiting Consultant Clinics,” Journal of the American Heart Association 5, no. 7 (2016): e002909, 10.1161/jaha.115.002909. 27364990 PMC5015359

[25] V. S. Baljepally and W. Metheny , “Rural‐Urban Disparities in Baseline Health Factors and Procedure Outcomes,” Journal of the National Medical Association 114, no. 2 (2022): 227–231, 10.1016/j.jnma.2022.01.001. 35109969

